# The innate immune protein calprotectin ablates the bactericidal activity of β-lactam antibiotics

**DOI:** 10.1073/pnas.2513462123

**Published:** 2026-01-14

**Authors:** Amanda Z. Velez, Jana N. Radin, Emily N. Kennedy, Joshua B. Parsons, Heather M. Tong, Emma Jung, Emily Alam, Lauren C. Radlinski, Nikki J. Wagner, Vance G. Fowler Jr., Sarah E. Rowe, Thomas Kehl-Fie, Brian P. Conlon

**Affiliations:** ^a^Department of Microbiology and Immunology, University of North Carolina, Chapel Hill, NC 27559; ^b^Department of Microbiology and Immunology, University of Iowa, Iowa City, IA 52242; ^c^Division of Infectious Diseases, Duke University School of Medicine, Durham, NC 27710; ^d^Department of Medical Microbiology and Immunology, School of Medicine, University of California, Davis, CA 95616

**Keywords:** calprotectin, antibiotic tolerance, *Staphylococcus aureus*, zinc, autolysis

## Abstract

β-lactam antibiotics are among the most widely used treatments for bacterial infections, yet therapeutic failure is common even when no genetic resistance is detected. Understanding how host factors influence antibiotic efficacy is critical for improving outcomes. Here, we identify a host-derived mechanism of antibiotic tolerance mediated by calprotectin (CP), a zinc-binding protein released in large quantities by neutrophils during infection. We show that CP induces tolerance to β-lactam antibiotics in *Staphylococcus aureus* by chelating zinc and inactivating autolysins, zinc-dependent enzymes required for cell wall degradation and bacterial lysis following β-lactam treatment. This protective effect was specific to β-lactam antibiotics at concentrations of CP showing minimal impact on bacterial growth or metabolic state. Mechanistic studies revealed that CP inhibits the autolytic activity of Atl, the major *S. aureus* autolysin, by depriving the enzyme of its zinc cofactor. In a murine infection model, the efficacy of oxacillin was significantly enhanced in CP-deficient mice, demonstrating that CP impairs β-lactam activity in vivo. These findings reveal a form of immune-mediated antibiotic tolerance driven by metal sequestration and suggest that zinc availability at infection sites plays a critical role in shaping treatment outcomes.

## Significance

Antibiotic failure is a major clinical problem that cannot always be explained by traditional resistance mechanisms. This study reveals that the host immune protein calprotectin, which is abundant at infection sites, can interfere with β-lactam antibiotics by sequestering zinc and inactivating bacterial autolysins that mediate cell lysis. This finding uncovers a mechanism of antibiotic tolerance that arises from the host response itself, rather than from the pathogen. Understanding how immune factors like calprotectin alter antibiotic activity opens the door to host-targeted strategies that enhance treatment outcomes. These findings suggest that local zinc availability during infection could be a critical, and previously underappreciated, determinant of β-lactam efficacy.

β-lactam antibiotics, such as penicillins and cephalosporins, are essential for the treatment of infections caused by a wide range of bacterial species (1). *Staphylococcus aureus* is the world’s leading bacterial cause of death (2). β-lactam antibiotics are the treatment of choice for infections caused by methicillin-susceptible *S. aureus* (MSSA) and cephalosporins such as ceftobiprole are used to treat methicillin-resistant *S. aureus* (MRSA) infection (3, 4). Treatment failure for *S. aureus* bloodstream infection, even when the appropriate antibiotic therapy is applied, remains common (5). Antibiotic tolerance, defined as bacterial survival in the presence of an ordinarily lethal antibiotic concentration without an associated change to the minimum inhibitory concentration (MIC), is thought to contribute to treatment failure (6). While tolerant bacteria, by definition, cannot grow in the presence of antibiotics, their increased survival necessitates prolonged antibiotic regimens and can result in treatment failure and infection recurrence once antibiotic pressure is lifted. The host conditions and factors that lead to β-lactam tolerance during infection are poorly understood.

β-lactams covalently bind to bacterial penicillin-binding-proteins, inhibiting the polymerization and cross-linking of peptidoglycan to reduce cell wall integrity (7). Autolysins are lytic enzymes functioning generally as an amidase, glycosidase, or endopeptidase, depending on the targeted bond within the cell wall (8). Autolysins function in opposition to penicillin-binding proteins, maintaining the balance between cell wall degradation and synthesis. Following β-lactam inhibition of peptidoglycan biosynthesis, bacterial autolysin activity leads to cell wall degradation, lysis, and death (9–11).

Here, we investigated the effect of a major mammalian innate immune effector, calprotectin (CP), on β-lactam treatment efficacy. CP accounts for approximately 40% of neutrophil cytoplasmic protein content, and it accumulates extracellularly to mg/mL concentrations at the site of infection (12). A heterodimer of S100A8 and S100A9, CP tightly binds first-row transition metals, including zinc, manganese, iron, copper, and nickel (13–16). This activity contributes to the ability of the host to impose metal starvation on invading pathogens representing an important part of nutritional immunity (17, 18). Imaging studies have revealed staphylococcal abscesses are highly metal-restricted and that CP is present in high abundance (17, 19). In multiple models, the loss of CP increases the ability of pathogens to obtain metals and activate metal-dependent processes (18–24). This study reveals that host-imposed zinc limitation ablates autolysin activity, dramatically reducing the killing activity of β-lactam antibiotics in vitro and in a murine bacteremia infection model.

## Results

### The Presence of Calprotectin Negatively Impacts the Efficacy of β-Lactam Antibiotics.

**Fig. 1.**
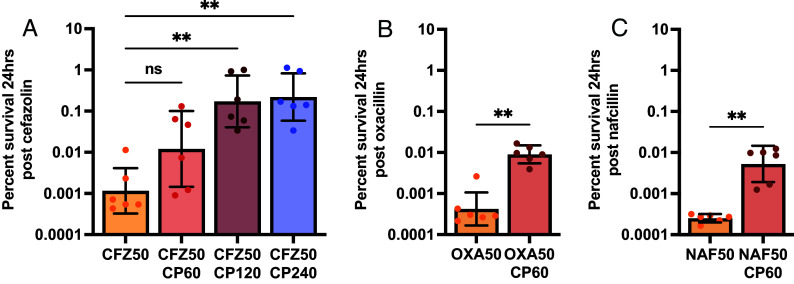
Calprotectin negatively impacts the efficacy of cell-wall acting antibiotics. *S. aureus* Newman was initially grown to mid-exponential phase in 38% TSB and 62% CP buffer. Cultures were then either treated with (*A*) cefazolin (CFZ50) at 50 μg/mL and CP at 60, 120, or 240 μg/mL; (*B*) oxacillin (OXA50) at 50 μg/mL with 60 μg/mL CP, or (*C*) nafcillin (NAF50) at 50 μg/mL with 60 μg/mL CP. Graphed is the percent survival presented on a log scale after 24 h treatment. Bars show the means with error bars representing SD. All experiments were completed on 3 separate days using two independent cultures (N = 6). Statistical significance (*P* ≤ 0.05) was determined by Kruskal–Wallis with Dunn’s multiple comparison to the control group of CFZ50 (*A*) or by Mann–Whitney test (*B* and *C*).

As β-lactams are a first-line treatment for *S. aureus* infections and CP is present in high abundance within the abscess, how CP influences the treatment efficacy of cefazolin against the MSSA strain Newman was first examined. Following growth to mid-exponential phase in TSB-based media, a range of physiologically relevant CP concentrations was added alongside cefazolin. Interestingly, the addition of CP promoted increased staphylococcal survival in a dose-dependent manner (Fig. 1*A*) with no impact on bacterial growth (*SI Appendix*, Fig. S1*A*). To determine if tolerance in the presence of CP was specific to cephalosporins or generalizable across different subclasses of β-lactam antibiotics, the impact of CP on the antistaphylococcal penicillins was next investigated. In the presence of 60 μg/mL CP, approximately 50-fold more *S. aureus* survived treatment with both oxacillin and nafcillin (Fig. 1 *B* and *C* and *SI Appendix*, Fig. S1 *B* and *C*). When oxacillin concentrations at 0.1X, 1X, and 2X the MIC were tested, the presence of CP was found to decrease antibiotic efficacy for all concentrations examined above the MIC (*SI Appendix*, Fig. S2). Furthermore, the induction of antibiotic tolerance with the addition of CP was found to be specific to β-lactam antibiotics, as the addition of 60 μg/mL CP had no effect on ciprofloxacin efficacy tested at 3X and 20X the MIC (*SI Appendix*, Fig. S3 *A* and *B*). To explore if changes in antibiotic efficacy were specific to the MSSA strain Newman, multiple clinical isolates of *S. aureus* (*SI Appendix*, Table S1) were tested and a similar induction of antibiotic tolerance in all isolates was observed (*SI Appendix*, Fig. S4*A*). Additionally, the presence of CP similarly increased tolerance of a clinical MRSA bacteremia isolate and the MRSA laboratory strain JE2 (*SI Appendix*, Table S1) to ceftobiprole, a β-lactam approved for the treatment of MRSA bacteremia (*SI Appendix*, Fig. S4*B*).

To examine the possibility that general protein binding was driving antibiotic inefficacy, antibiotic killing in bovine serum albumin (BSA) at similar protein concentrations to CP was examined. Our results showed that BSA had no impact on survival postoxacillin treatment (*SI Appendix*, Fig. S4*C*). Additionally, the observed increased survival against antibiotics in the presence of CP was not associated with a change in the MIC of the antibiotic (*SI Appendix*, Table S2), demonstrating that antibiotics remain unbound and capable of inhibiting their target in the presence of CP. Together, our results show that CP dramatically increases survival against the main class of antibiotics used in the treatment of *S. aureus* infections, with no detectable change to the MIC. Furthermore, this effect was observed across multiple β-lactam antibiotics and across multiple *S. aureus* strains, both MSSA and MRSA.

### Zinc Limitation Induces Antibiotic Tolerance in a Mechanism Independent of Target Site Activity or Metabolic State.

**Fig. 2.**
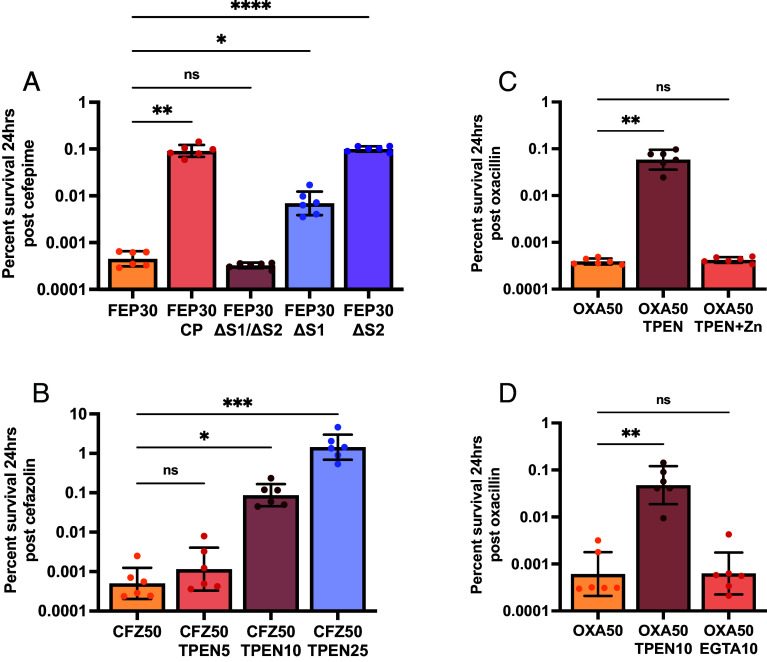
Zinc limitation induces antibiotic tolerance. *S. aureus* Newman was grown to mid-exponential phase in 38% TSB and 62% CP buffer. Cultures were then treated with (*A*) cefepime at 30 μg/mL (FEP30) and either wildtype CP (240 μg/mL), ΔS1/ΔS2 (240 μg/mL), ΔS1 (480 μg/mL), or ΔS2 (480 μg/mL). Single site mutant concentrations were doubled as they have half the binding capacity of wildtype CP; (*B*) cefazolin at 50 μg/mL (CFZ50) and increasing concentrations of TPEN, 5 μM to 25 μM; (*C*) oxacillin at 50 μg/mL (OXA50) and 10 μM TPEN with and without the addition of 50 μM ZnSO_4_; or (*D*) oxacillin at 50 μg/mL (OXA50) and 10 μM TPEN or 10 μM EGTA. Data graphed represent the mean percent survival 24 h posttreatment on a log scale with error bars showing SD. Each experiment was completed on 3 separate days using two independent cultures (N = 6). Statistical significance (*P* ≤ 0.05) was determined by Welch’s ANOVA with Dunnett’s T3 multiple comparisons test (*A* and *C*) or by Kruskal–Wallis with Dunn’s multiple comparisons test (*B* and *D*).

CP sequesters zinc and manganese via two distinct metal-binding sites located at the dimer interface of the S100A8 and S100A9 subunits: S1, a His_6_ motif and S2, a His_3_Asp motif. A CP variant that lacks both metal binding sites and cannot bind transition metals was next examined for its capacity to induce antibiotic tolerance (13). The mutant protein was unable to induce tolerance to oxacillin, strongly implicating metal limitation as the driver of β-lactam tolerance. (Fig. 2*A* and *SI Appendix*, Fig. S5*A*).

To identify which metal was responsible for reducing antibiotic efficacy, single site disruptions for metal binding (ΔS1 and ΔS2) were examined (13). S1 binds both zinc and manganese while S2 binds only zinc. If zinc sequestration is driving β-lactam tolerance, then the single mutants will retain the ability to induce tolerance, whereas if manganese limitation is driving the phenotype, the S1 mutation alone should be sufficient to ablate the induction of tolerance. Comparison between the two site mutants suggested CP decreases antibiotic efficacy primarily through the limitation of zinc as both variants significantly increase bacterial survival postcefepime treatment (Fig. 2*A* and *SI Appendix*, Fig. S5*A*).

To confirm that zinc limitation induces β-lactam tolerance, antibiotic killing in the presence of the potent zinc chelator, N, N, N′, N′-tetrakis(2-pyridinylmethyl)-1,2-ethanediamine (TPEN) was examined. Similar to CP, the addition of TPEN resulted in decreased efficacy of β-lactam antibiotics in a dose-dependent response, culminating in a 1,000-fold increase in *S. aureus* survivors relative to the metal replete control (Fig. 2*B* and *SI Appendix*, Fig. S5*B*). Furthermore, supplementation of the media containing both TPEN and exogenous ZnSO_4_ was sufficient to restore antibiotic efficacy (Fig. 2*C* and *SI Appendix*, Fig. S5*C*). When EGTA, a potent chelator of calcium with limited affinity for zinc, was used, no change to antibiotic killing kinetics as compared to the chelator absent control was observed (Fig. 2*D* and *SI Appendix*, Fig. S5*D*). Together, these data demonstrate that zinc limitation leads to the development of tolerance to β-lactam antibiotics.

**Fig. 3.**
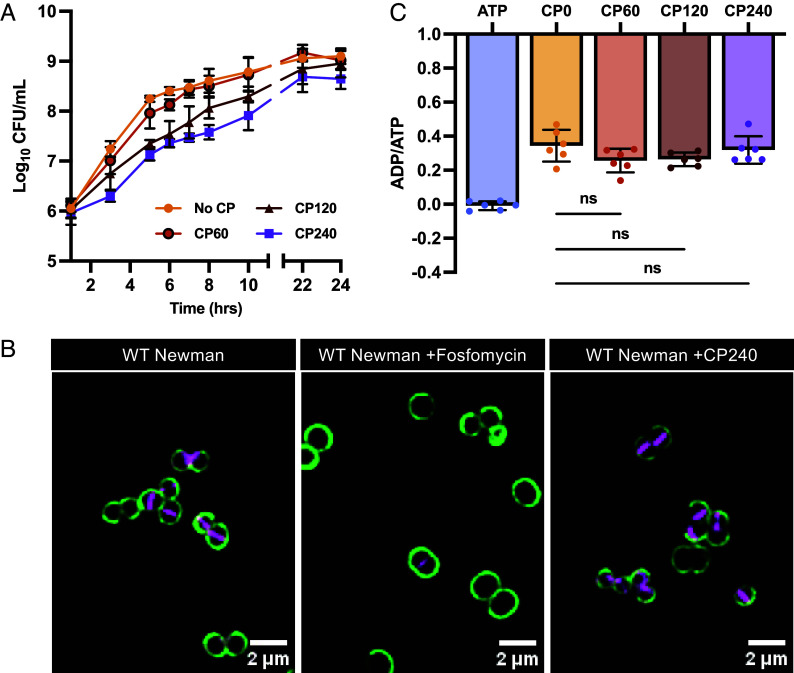
Zinc limitation induces antibiotic tolerance in a mechanism independent of target site activity or metabolic state. (*A*) Growth curve of *S. aureus* Newman in 38% TSB and 62% CP buffer containing CP at concentrations ranging from 0 to 240 μg/mL. This experiment was completed on 2 separate days using three independent cultures (N = 6). (*B*) *S. aureus* Newman was grown in 38% TSB and 62% CP buffer to mid-exponential phase and then either left untreated or treated with CP (240 μg/mL) or fosfomycin (50 μg/mL) for 1 h. Cells were then stained with HADA followed by WGA-488 and representative cells of the population are presented for each condition. (*C*) At ~10^6^ CFU/mL, ADP/ATP ratios of cells growing in the presence of CP (0 to 240 μg/mL) were determined using a luciferase-based quantification kit. Measurement of ATP (1 μM) functions as the positive control. This experiment was completed on 3 separate days using two independent cultures (N = 6). Statistical significance (*P* ≤ 0.05) was determined by Welch’s ANOVA with Dunnett’s T3 multiple comparison to the absence of CP condition (CP0).

Antibiotic tolerance has frequently been associated with slower growth, decreased metabolism, and reduced target activity. However, the effect of CP on β-lactam survival was observed at a concentration twofold lower than the IC50 for CP in these conditions (13). Consistent with this, the CFU recovered in the presence of 60 μg/mL CP was similar to media lacking CP (Fig. 3*A*). This strongly suggests that CP-induced tolerance, particularly at lower concentrations, is not attributable simply to slowed growth. However, this does not eliminate the possible effect of growth delay on a subset of cells which could additionally contribute to the development of tolerance. To examine cell-wall biosynthesis activity, the fluorescent d-amino acid (HADA) which accumulates at sites of cell wall synthesis and a cell surface carbohydrate stain (wheat germ agglutinin conjugated to Alexa Fluor 488), were used to visualize regions of active cell wall synthesis. These results showed that the addition of CP did not result in the inhibition of cell wall synthesis, as seen by the accumulation of HADA signal comparable to that of untreated cells (Fig. 3*B* and *SI Appendix*, Fig. S6). This suggests that zinc limitation induces antibiotic tolerance by a mechanism independent of cell wall synthesis inhibition.

To examine the energy state of the cell in the presence of CP, the EnzyLight ADP/ATP Ratio Assay Kit was utilized, measuring ATP and ADP levels through a luminescent reaction with the luciferase enzyme. Measurement of cellular ADP/ATP ratios with and without the addition of CP showed no reductions to the energy equilibrium of the cell (Fig. 3*C*). These results suggest that CP-induced tolerance is independent of a reduction in metabolic activity.

### Bacterial Autolytic Activity is Decreased by the Zinc-limiting Activity of Calprotectin.

Because growth, target site activity or metabolic state are not decreased in the presence of CP, tolerance must be induced through an alternative mechanism. β-lactam antibiotics inhibit cell wall synthesis by covalently binding to transpeptidases or penicillin-binding proteins. This inhibition prevents the cross-linking of peptidoglycan chains, weakening the cell wall. The resulting cell lysis is thought to be mediated by autolysins, which are cell wall degrading enzymes. Supporting this model, multiple studies have shown that deletion of autolysin genes enhances bacterial survival following β-lactam treatment (9–11). The genome of *S. aureus* encodes for multiple autolysins, four of which require zinc as a cofactor for catalytic activity, including Atl, the primary *S. aureus* autolysin. To examine the requirement of zinc for autolytic activity, parallel zymography was performed in the presence and absence of the zinc-chelator, TPEN. Zymography is a technique used to visualize autolytic activity by examining regions of clearing in a polyacrylamide gel embedded with bacterial cell wall. Comparison of gels with and without TPEN showed that the addition of TPEN inhibited Atl-mediated cell wall clearance (*SI Appendix*, Fig. S7 *A* and *B*).

**Fig. 4.**
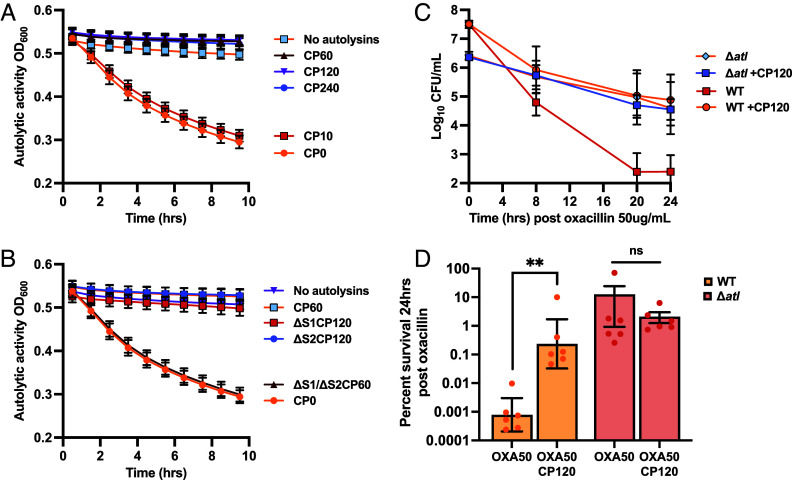
Autolytic activity is decreased by the zinc-limiting activity of calprotectin. (*A*) *S. aureus* Newman was grown in 38% TSB and 62% CP buffer overnight prior to pelleting the cells and combining the supernatant with heat-killed *S. aureus* RN4220 cells and increasing concentrations of CP (0 to 240 μg/mL). (*B*) Cell supernatant was combined with heat-killed *S. aureus* RN4220 cells in the presence of wild-type CP (60 μg/mL), ΔS1/ΔS2 (60 μg/mL), ΔS1 (120 μg/mL), or ΔS2 (120 μg/mL). *S. aureus* Newman wild-type or Δ*atl* was incubated for 3 h in 38% TSB and 62% CP buffer and then challenged with oxacillin at 50 μg/mL with and without 120 μg/mL CP. Surviving cells were enumerated at 8, 20, and 24 h posttreatment (*C*) with percent survival calculated at 24 h (*D*). Each experiment was completed on 3 separate days using two independent cultures (N = 6). Statistical significance (*P* ≤ 0.05) was determined by Mann–Whitney test between conditions with and without CP for each strain.

To examine if CP can reduce autolysis through zinc limitation, the supernatant of bacterial cultures was combined with heat-killed *S. aureus* cells and autolytic activity was assessed. Active autolysins released extracellularly will lyse heat-killed *S. aureus,* resulting in a decrease in OD_600_ over time. Clearance in OD_600_ is due to the activity of autolysins, particularly that of Atl, as supernatant collected from Δ*atl* cultures shows a lack of activity, as evident by limited change in the OD_600_ (*SI Appendix*, Fig. S7*C*). In the presence of CP, complete loss of autolytic activity was observed as represented by little change in OD (Fig. 4*A*). When CP variants were used, the ΔS1/ΔS2 mutant, but not the ΔS1 or ΔS2 mutant, both which retain zinc binding, resulted in complete recovery of autolytic activity (Fig. 4*B*). Since both sites can bind zinc, recovery of autolytic activity for only ΔS1/ΔS2 implies that zinc limitation is responsible for the lack of autolytic activity in the presence of CP.

To determine if autolytic activity was responsible for the induction of tolerance in the presence of CP, mutation in a gene encoding the zinc-dependent autolysin, *atl,* was compared to wildtype. Both Δ*atl* and wildtype cultures were challenged with oxacillin, and surviving cells were monitored over a period of 24 h (Fig. 4*C*). In the absence of *atl,* the addition of CP failed to exhibit a significant effect on antibiotic survival (Fig. 4*D*). This suggests that inhibition of autolysin activity may contribute to the development of tolerance in the presence of CP. To visualize potential changes in the cell wall of *S. aureus* because of decreased autolytic activity in the presence of CP, cell wall structure was also examined by transmission electron microscopy. The addition of 60 μg/mL CP showed noticeable changes to the structure of the cell walls after 2 h (*SI Appendix*, Fig. S8). Comparison between cross-sections showed regions of increased peptidoglycan for CP-treated cells (black arrows). This is reminiscent of previous observations for Δ*atl S. aureus,* where the absence of Atl activity leads to thread-like peptidoglycan interconnections between cells (25).

### The Presence of Calprotectin Negatively Impacts the Efficacy of Cell-Wall Acting Antibiotics During Bacteremia.

**Fig. 5.**
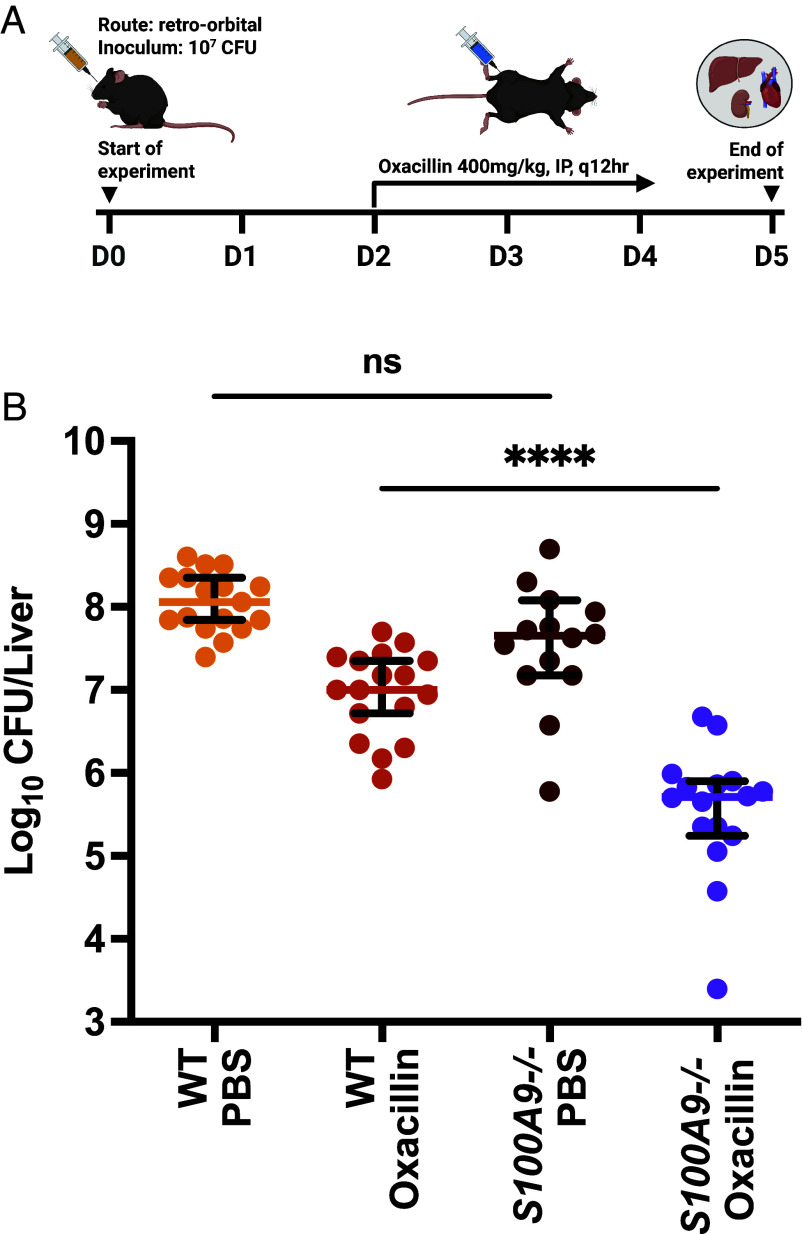
The presence of calprotectin negatively impacts the efficacy of cell-wall-acting antibiotics in vivo. (*A*) Timeline of infection for the murine bacteremia model. Wild-type and S100A9^−/−^ C57BL/6 mice were infected retro-orbitally with 10^7^ CFU of *S. aureus* Newman. After 48 h, oxacillin intraperitoneal treatment at 400 mg/kg administered every 12 h was initiated and continued over 3 d. At 5 d postinfection, mice were killed and select internal organs were homogenized, diluted, and plated to determine bacterial burden. (*B*) Surviving bacteria within the liver of infected mice at day 5 postinfection. Data graphed are the median with 95% CI. This experiment was completed twice with each data point representing an individual mouse. Statistical significance was determined by Welch’s ANOVA with Dunnett’s T3 multiple comparisons test. Results are considered significant if *P* ≤ 0.05.

As CP is a heterodimeric S100 protein formed by association of monomers S100A8 and S100A9, mice that are *S100A9^−/−^* are essentially deficient in CP (26). To determine the impact of CP on the in vivo activity of cell-wall targeting antibiotics, both wildtype and *S100A9^−/−^* mice were infected with *S. aureus* Newman retro-orbitally and then treated with oxacillin (Fig. 5*A*). Oxacillin showed significantly improved bacterial clearance in the liver of CP-deficient mice with approximately 20-fold less survivors relative to wild-type mice (Fig. 5*B*). These effects were not observed in the kidney and heart tissue where no significant differences in oxacillin treatment between wild-type and *S100A9^−/−^* mice were observed (*SI Appendix*, Fig. S9 *A* and *B*). Together, these results demonstrate that CP induces potent tolerance to β-lactam antibiotics in *S. aureus*, resulting in reduced β-lactam efficacy in vitro and in vivo.

## Discussion

The treatment of *S. aureus* infections is characterized by a high incidence of failure, even in cases involving drug-susceptible strains (27, 28). Several studies have shown that antibiotic efficacy is dramatically modulated by the host (29–33). Understanding how antibiotics function within the infectious environment is key to preserving and enhancing their efficacy. This study investigated how CP, one of the major proteins of the innate immune system, influences the efficacy of β-lactam antibiotics against *S. aureus* infection. Our results show that CP-driven zinc chelation induces tolerance to β-lactam antibiotics and inhibits zinc-dependent autolysins. Furthermore, CP significantly inhibits oxacillin efficacy in a mouse model of bacteremia.

CP is an abundant protein within the neutrophil cytosol and at sites of infection, the influx of neutrophils leads to the accumulation of CP in excess of 1 mg/mL (12). The metal-binding ability of CP plays a key role in nutritional immunity, the process by which essential nutrients for microbial survival are limited by the host. While CP binds multiple first row transition metals tightly (13–16), the current results strongly support that zinc limitation induces tolerance to β-lactam antibiotics. The contribution of CP to metal limitation varies by tissue. However, CP is not the only zinc binding protein found at sites of infection, with humans producing both S100A7, secreted by keratinocytes, and S100A12, secreted by neutrophils, as well as other unidentified mechanisms for restricting zinc availability (17, 19, 34, 35). These zinc withholding mechanisms could all contribute to reduced β-lactam efficacy within the host, affecting the impact that loss of CP has on antibiotic tolerance. Despite these additional mechanisms, loss of CP does increase the availability of zinc (23, 24), particularly in the liver, as evidenced by the increased virulence of an *Acinetobacter baumannii* zinc-transporter mutant in the liver of CP-deficient mice (18). This suggests that the increased efficacy of β-lactams observed in the liver of CP-deficient mice in this study is driven by increased zinc availability.

Even in the presence of high levels of CP, *S. aureus* is well equipped to transport zinc and maintain sufficient levels for intracellular functionality (26, 36). However, extracellular zinc-dependent enzymes are vulnerable to CP sequestration of zinc. In support of this, CP can potently inhibit the zinc-dependent protease activity of *Pseudomonas aeruginosa* extracellular virulence factors LasA and LasB (37). Additional *S. aureus* metalloproteases may also be vulnerable to metal limitation by CP. The extracellular zinc metalloprotease, aureolysin, which cleaves host proteins and antimicrobial peptides, functions as an important virulence factor for *S. aureus* (38), suggesting that CP may influence *S. aureus* pathogenesis as well as antibiotic susceptibility.

Numerous autolysins over a broad range of Gram-positive and Gram-negative species are extracellular metalloenzymes requiring zinc for autolytic activity, including *Clostridioides difficile* Cwp6, *Streptococcus pneumoniae* LytA, *Helicobacter pylori* Csd2, and *Escherichia coli* AmiA/B/C, among many others (39–42). Thus, the role of zinc limitation in altering cell wall homeostasis remains to be investigated in depth and may prove to be a major influence on autolytic functioning for multiple pathogens.

Additional zinc-dependent extracellular enzymes capable of influencing antibiotic treatment outcomes include metallo-β-lactamases. Previous research has shown that the addition of TPEN was sufficient to restore antibiotic susceptibility below the clinical breakpoint for a carbapenem resistant clinical isolate of *A. baumannii* expressing a zinc-dependent carbapenemase (18). Inactivation of extracellular zinc-dependent enzymes therefore likely represents an important function of CP during infection, albeit with major negative consequences for β-lactam efficacy.

The levels of transition metals available to pathogens at the site of infection is also dependent on dietary intake and can be manipulated by diet modification (43). Increased manganese in the diet has been found to increase the availability of this metal during *S. aureus* infection (44). Therefore, it is interesting to consider the possibility that a high zinc diet could increase autolytic activity within the abscess. Although the ablation of autolysin activity is sufficient to induce significant tolerance to β-lactams, CP may also impact antibiotic tolerance through inhibition of other zinc and manganese-dependent processes that impact cell growth, particularly at higher concentrations.

In summary, these results show that CP dramatically increases survival against the main class of antibiotics used in the treatment of *S. aureus* infections. This study provides the first demonstration of a host-induced state of antibiotic tolerance mediated by targeted inactivation of a bacterial enzyme. While antibiotic tolerance has traditionally been attributed to metabolic downregulation or growth arrest, our findings suggest that a broader spectrum of host–pathogen interactions can substantially alter antibiotic efficacy. These mechanisms likely act in parallel, such that at concentrations of CP that slow bacterial growth, autolysin inhibition further enhances tolerance. Collectively, these effects highlight an underexplored dimension of antibiotic susceptibility that could be therapeutically targeted to improve treatment outcomes in patients.

## Methods

### Growth Conditions, Strains, and Reagents.

For all experiments unless otherwise clarified, *S. aureus* was grown overnight in 5 mL of Tryptic Soy Broth (TSB) and then diluted the following day in 3 mL of 38% TSB and 62% of a high-calcium buffer (20 mM Tris-HCl, 3 mM CaCl_2_, 100 mM NaCl, pH 7.5) (CP buffer). All bacterial growth was accomplished using a roller drum at 37 °C in closed-cap 15 mL conical tubes. Construction of Δ*atl* in the Newman *S. aureus* background was completed by transducing the single mutant from the Nebraska transposon Mutant Library (NTML) as previously described (45). Mutations were confirmed by antibiotic resistance to erythromycin and by PCR, using primers CCCTGCTATTGTCCAACCAA for *atl* and the transposon-specific primers as provided by NTML. Recombinant CP lacking cysteine residues to prevent disulfide bond formation was expressed and purified as previously described (46). To avoid the use of an ampicillin resistance cassette which encodes for a β-lactamase that was found to purify alongside CP, the expression vector was modified to include only a kanamycin resistance cassette.

### Timed Kill Curve Assay.

Overnight cultures of *S. aureus* were diluted 1:1,000 and incubated to reach mid-exponential phase, between 2 and 3 h depending on the strain of *S. aureus* utilized in each experiment. Following determination of the starting bacterial burden (0 h timepoint), cultures were then treated simultaneously with antibiotic and either CP, BSA, TPEN, or EGTA, at concentrations specified in each figure legend. Calcium was directly added to purified aliquots of CP at 3 mM CaCl_2_ prior to use. All cultures were then monitored for survival over a period of 24 h and the CFU/mL at timepoints 0, 8, 20, 24 h were examined. At each timepoint, a 100 μL aliquot was washed twice in PBS, diluted, and plated on Tryptic Soy Agar (TSA) plates. Colony-forming units (CFU) were enumerated following incubation at 37 °C overnight and graphed as bacterial survival over time. Data graphed as percent survival was calculated using the following formula: % survival = (24 h/0 h) × 100.

### Cell Wall Imaging Using Fluorescence Microscopy.

Imaging of the bacterial cell wall using fluorescence microscopy was accomplished as previously described (47). *S. aureus* Newman was back-diluted 1:1,000 and incubated to reach 10^7^ CFU/mL. Select cultures were then left untreated or treated with CP at 240 μg/mL or fosfomycin at 50 μg/mL for 1 h. Cell walls were then stained with 250 μM fluorescent D-amino acid HADA (Tocris Bioscience) for 5 min, followed by washing and staining with wheat germ agglutinin conjugated to Alexa Fluor 488 (WGA-488, Invitrogen) at 2 μg/mL for an additional 5 min. Cells were then washed and fixed with 4% paraformaldehyde and 2 μL pipetted between a glass coverslip and an agarose pad. Z-stack images of representative cells were then acquired using the Zeiss LSM900 microscope using the 63×/1.4 oil plan apo objective. Images were deconvolved using AutoQuant and processed with identical display adjustments using FIJI.

### Growth Curve Assay.

Overnight cultures of *S. aureus* Newman were diluted 1:1,000 in the presence of increasing concentrations of CP ranging from 0 to 240 μg/mL. Over a period of 24 h, 10 μL of culture was diluted and plated on TSA to determine CFU/mL.

### Measurement of Bacterial ADP/ATP Ratio.

The EnzyLight ADP/ATP Ratio Assay Kit (BioAssay Systems) was utilized according to the manufacturer’s instructions using cultures of *S. aureus* Newman grown to around 10^6^ CFU/mL in the presence of CP (0 to 240 μg/mL). Luminescence was recorded using white-opaque-walled 96-well plates and a BioTek Synergy H1 microplate reader (Agilent Technologies). Results were normalized to background signal and ATP (1 μM) served as the positive control for this assay.

### Autolysin Activity Assay.

Overnight cultures of *S. aureus* Newman grown in 38% TSB and 62% CP buffer were pelleted and the supernatant containing autolysins was collected and filter sterilized using a 0.22 μm filter. Aliquots of supernatant were then combined with CP at a range of concentrations (0 to 240 μg/mL) or combined with metal binding site disruptions, ΔS1/ΔS2 at 60 μg/mL, ΔS1 at 120 μg/mL, or ΔS2 at 120 μg/mL. For comparison, single-site mutant concentrations were doubled as they have half the binding capacity of wild-type CP. Following a 1 h incubation, 30 μL of supernatant was then combined with 70 μL of heat-killed *S. aureus* RN4220 resuspended in CP buffer in a 96-well plate. Over the course of 10 h, the OD_600_ was measured using a BioTek Synergy H1 microplate reader (Agilent Technologies) and plotted against time to examine autolytic activity.

### Murine Bacteremia Model.

In vivo infections were completed at the University of Illinois Urbana-Champaign following approval by the Institutional Animal Care and Use Committee (IACUC). Female wild-type C57BL/6 mice and CP-deficient mice (S100A9^−/−^ C57BL/6) were infected retro-orbitally with 10^7^ CFU of *S. aureus* Newman. The infection inoculum was prepared by backdiluting overnight cultures 1:100 in 5 mL of TSB. Cultures were then incubated using a roller drum at 37 °C in closed-cap 15 mL conical tubes to reach 10^8^ CFU/mL. Cells were pelleted, resuspended in 10 mL of PBS, and bacterial cell density was verified by dilution and plating on TSA. Mice were then inoculated with 10^7^ CFU by retro-orbital injection of 100 μL of bacterial suspension. At 48 h postinfection, antibiotic treatment was initiated at 400 mg/kg of oxacillin and administered intraperitoneally every 12 h over the course of 3 d. At the end of this treatment period, day 5 postinfection, the kidney, heart, and livers were harvested and homogenized to determine bacterial burdens.

## Supplementary Material

Appendix 01 (PDF)

## Data Availability

Excel files, prism files, and microscopy images have been deposited in Dataverse (https://doi.org/10.15139/S3/N3LTDZ) (48).
